# The role of vitamin D deficiency in the development and severity of oral lichen planus: a case-control study

**DOI:** 10.1007/s00784-025-06398-y

**Published:** 2025-05-30

**Authors:** Rania Shalaby, Ghada Nabil, Sally Ibrahim, Ali AW Kotb, Hatem Amer, Salsabeel Afifi

**Affiliations:** 1 Oral Medicine, Oral Diagnosis and Periodontology, Faculty of Dentistry, Fayoum University, Fayoum, Egypt; 2 Oral medicine, Faculty of Dentistry, Cairo University, Giza, Egypt; 3 Oral and Maxillofacial Pathology, Faculty of Dentistry, Fayoum University, Fayoum, Egypt; 4 Oral and Maxillofacial pathology, Faculty of Dentistry, Cairo University, Giza, Egypt; 5 6th October city, Egypt

**Keywords:** Serum vitamin D, Vitamin D deficiency, Oral lichen planus, Depression, Anxiety, Sun exposure, Severity

## Abstract

**Objectives:**

Examine the responsibility of Vitamin D (VD) deficit as a risk element in the development and severity of oral lichen planus (OLP) considering dietary habits, sex, sun exposure, socioeconomic class and psychological factors.

**Materials and methods:**

Blood samples from 35 OLP patients and 35 healthy controls were collected for the analysis of serum VD level (SVDL) by ELISA. Vitamin D deficiency was considered when SVDL was ≤ 20 ng/ml. Additionally, a structured questionnaire was used to analyze other possible confounders.

**Results:**

SVDL were statistically significant greater in the control group than the OLP group (*P* ≤ 0.001). There was difference between the 3 types of OLP with statistical significance in which the least values for SVDL were in erosive type (*P* ≤ 0.001). Furthermore, no statistically significant difference was found in SVDL between dysplastic and non-dysplastic lesions. In terms of VD deficiency, a statistically significant distinction was noticed between the two research sets (*P* ≤ 0.001) and was more pronounced in erosive and atrophic types than reticular types. Depression as well as sun exposure significantly affected number of patients having VD deficiency (*P* = 0.001, 0.027). Results revealed a statistically significant direct relation between SVDL and the OLP (odds ratio = 1.38; 95%CI = 1.18–1.617).

**Conclusion:**

VD deficiency plays a vital role in OLP and the development of more severe forms.

**Clinical relevance:**

It corroborates that VD deficiency is a probable risk factor of OLP and the development of more severe forms considering many confounders.

**Supplementary Information:**

The online version contains supplementary material available at 10.1007/s00784-025-06398-y.

## Introduction

Oral lichen planus (OLP) is a common, chronic, immunologic disease with a worldwide prevalence of 1.01% [1]. Clinically, it is diagnosed by the existence of lace-like white lesions, either with or without erosive or atrophic zones and less commonly appear as plaque-like, papular, or bullous lesions [2]. To date, the etiopathogenesis of OLP is still unidentified. Nevertheless, it is believed to be triggered by a dysregulation of cell-mediated immunity, in which keratinocyte apoptosis is caused by auto-reactive cytotoxic T-cells (CD8) [2, 3].

In recent years, the relationship between vitamin D (VD) and immunologically mediated diseases has drawn increasing attention. VD, a fat-soluble vitamin, exerts its action through VD receptors (VDR), which are abundantly present on T-lymphocytes. There is emerging evidence about the role of active VD (25-hydroxy VD3) in the control of immune reactions. This includes down-regulation of T-helper (CD4) cells and inhibition of B-cell differentiation, as well as restriction of antigen presentation while upregulating regulatory T-cells [4–10]. In this context, previous studies have suggested VD deficiency as a possible contributing element in the occurrence of OLP [11–13]. Moreover, it has been proposed as a possible preventive and curative agent for OLP [9, 10, 14–16].

Despite the WHO having classified OLP as a potentially malignant lesion, González-Moles et al. [17, 19] asserted that the malignant development potential of OLP is undervalued [2, 17]. This is ascribed to the diagnostic criteria proposed by Van der Meij and Van der Waal, which imply the exclusion of the OLP diagnosis when dysplasia is evident [18]. Consequently, several studies recommend against using epithelial dysplasia as a criterion for rejecting the diagnosis of OLP, as it may manifest with the progression of the disease [17, 19].

To date, the responsibility of serum vitamin D level (SVDL) as a possible risk element in the OLP development and its advancement into oral squamous cell carcinoma is unclear. Furthermore, a recent meta-analysis revealed that OLP participants experience a greater incidence of stress, anxiety, and depression [17]. For these reasons, the aim of the present study was to compare SVDL between OLP patients and healthy controls as well as between OLP subtypes representing different severities of the condition. In addition, the current study compared SVDL in OLP lesions that showed signs of dysplasia in histopathologic examination (Dysplastic) and those with no signs of dysplasia (non-dysplastic) to ascertain whether VD deficiency ought to be regarded as a contributing factor for the onset, progression, to more severe forms, or probably malignant transformation of OLP. Additionally, psychological assessment utilizing the Depression Anxiety and Stress Scale (DASS-21 scale) [18], and socioeconomic status using Kuppuswamy’s scale [19] was performed.

## Subjects and methods

### Study design

Observational case-control study that followed the STROBE guidelines.

### Study settings and ethical considerations

All eligible subjects were enlisted from the treatment centers of the Faculty of Dentistry at Fayoum University and Al Ahram Canadian University, Egypt from July to October 2023. Before the investigation started, each participant signed an informed consent form. This study conforms to all the principles of the Helsinki Declaration. It was permitted (serial number: IRB00012891#78) by the authors’ Institutional Review Board (The Ethical Committee of the Faculty of Dentistry, Al Ahram Canadian University).

### Participants

Seventy participants were assigned into two groups. Thirty-five patients in Group A had a clinical picture that matches the clinical diagnosis of OLP as stated by Warnakulasuriya et al. [2] and histological findings that confirm the diagnosis, mainly liquefaction degeneration of the basal cells and a band-like area of lymphocytic infiltration limited to the upper part of connective tissue, without exclusion of dysplasia as recommended by González-Moles et al. [20]. Clinically, patients were classified into three main types: reticular (including papular or plaque type), atrophic, and bullous erosive ulcerative OLP. Histologically, any signs of epithelial dysplasia were recorded, so lesions were further divided into dysplastic and non-dysplastic OLP. Group B included 35 healthy volunteers which were matched by sex, age, and dietary habits (whether they were vegetarian or non-vegetarians).

Exclusion criteria involved:

1. Patients receiving any medication including corticosteroids for the management of OLP or any other condition for the past 6 months.
2. Individuals who have been using multivitamins, different types of VD supplements, or medications that might alter VD measures as discussed by Gröber and Kisters 2012 [21].
3. Patients with oral lichenoid lesions including lesions caused by contact with amalgam, reaction to drug or in the setting of graft versus host disease [22].
4. Patients who smoke and/or use smokeless tobacco because additional keratotic lesions caused by tobacco products could mislead the examiners [20].
5. Pregnant females or individuals with any systemic medical condition.
6. When VD ≥ 100 ng/ml as it is considered VD toxicity.

To gather information, each participant filled out a structured questionnaire. Age, sex, country, early living circumstances (rural or urban), systemic medical conditions, history of drug intake, consumption of VD supplements, dietary habits, psychological assessment applying the DASS-21 scale [18], and socioeconomic status using Kuppuswamy’s scale [19] were among the details it contained.

### Measurement of index test (SVDL ng/ml)

After patients’ enrollment and signing an informed consent, investigators collected 5 ml of peripheral venous blood from all participants. For the quantification of VD3, the enzyme-linked immunosorbent assay (ELISA) (kit supplied by ORGENTEC Diagnostika GmbH, Germany) was employed to analyze all samples concurrently in accordance with the manufacturer’s instructions. A competitive ELISA-based technique was performed to measure the level of 25-OH VD3/D2 in blood samples.

According to the Society of Clinical Endocrinology [23], the blood level of 25(OH)D above 20 ng/ml is considered sufficient to prevent rickets and osteomalacia in children and adults respectively, with normal being between 30 and 100 ng/ml, deficiency between 12 and 19 ng/ml and serious deficiency < 11 ng/ml. In the present study, SVDL ≤ 20 ng/ml were assigned to the VD deficiency set, while levels ranging between 20 and 100 ng/ml were classified as VD sufficiency, while levels ≥ 100 ng/ml were considered VD toxicity [23, 24].

### Measures to control potential bias

Efforts to control potential sources of bias were as follows: (1) To minimize selection bias and confounders, OLP patients as well as age- and sex-matched controls were enrolled in a consecutive pattern and with comparable dietary habits; (2) Blinding: blood samples were sent to the lab for VD measurement marked as group A and B and not as case and control; also, the main investigator (A.S.) who performed the clinical examination and patient enrollment was blinded to VD measurements. The primary investigator (S.R.) collected all data and performed the statistics.

### Sample size Estimation

Based on prior research [4] that explored SVDL in OLP patients versus normal healthy controls and using proportion of inequality in two independent groups (Fisher’s exact test), calculations generated the need for 70 participants (35 in each group, case and control) to achieve 0.05 statistical significance (α-error) and a 90% power (1-β). G*Power 3.1.9.7 was utilized to measure the sample size.

### Statistical analysis

Using the Jamovie application, Shapiro-Wilk tests were used for exploring data normality and Levene’s test was applied for homogeneity of variances. For parametric continuous data, the student-t test (two independent groups) and one-way ANOVA (> two groups) were followed by a post-Hoc Test. For non-parametric data, the Mann-Whitney test was applied to contrast two independent groups, and the Kruskal-Wallis test was employed to assess more than two independent groups, followed by Dwass-Steel-Critchlow-Fligner pairwise comparisons. A regression analysis was performed to correlate SVDL (predictor) and presence or severity of OLP (outcome).

## Results

**Fig. 1:**
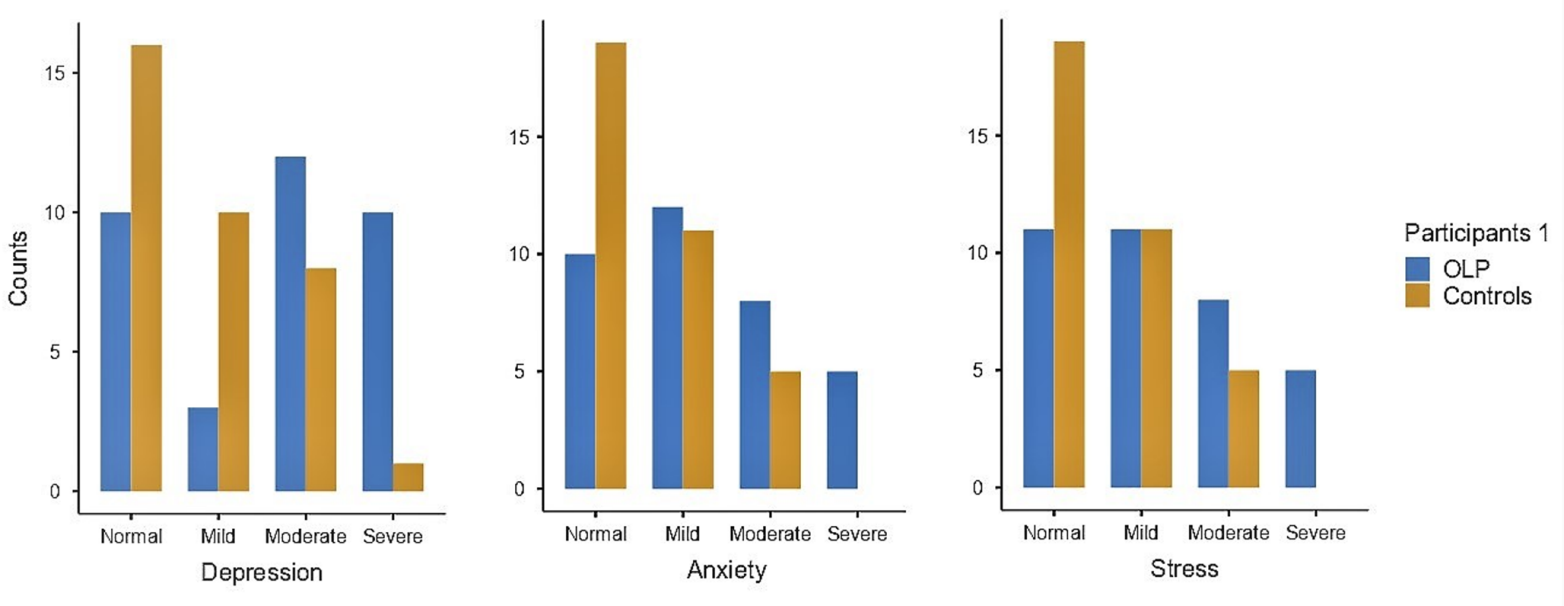
DASS-21 score in OLP patients versus controls

The present study included 70 Egyptian participants involving 35 OLP participants and 35 healthy volunteers. Participants’ characteristics are displayed in Table 1 in detail. According to Kuppuswamy’s socioeconomic status scale, there was a statistically significant difference between OLP and the healthy controls (*P* = 0.028); while, no significant difference was noted among different types of OLP cases (*P* = 0.895). It was found that the majority of the OLP group (45.7%) was of middle level compared to the control group (28.6%) while the majority of control group was of upper middle level (57.1%). Regarding results of the DASS-21 scale, a statistically significant greater number of patients having depression, anxiety and stress in OLP group than in control group (*P* = 0.004, 0.036, and 0.05, respectively). Moreover, there were statistically significant differences in number of patients having depression, anxiety and stress between the three types of OLP (*P* = 0.05, 0.021 and 0.011 respectively). It was noticed that severe depression occurred in 50% of erosive OLP and 50% of atrophic OLP compared to none of those with reticular type. Similarly, severe anxiety and stress occurred in 100% of participants with erosive OLP, while none of those with atrophic or reticular lesions had severe anxiety or stress as shown in Table 1 and Fig. 1.

**Fig. 2:**
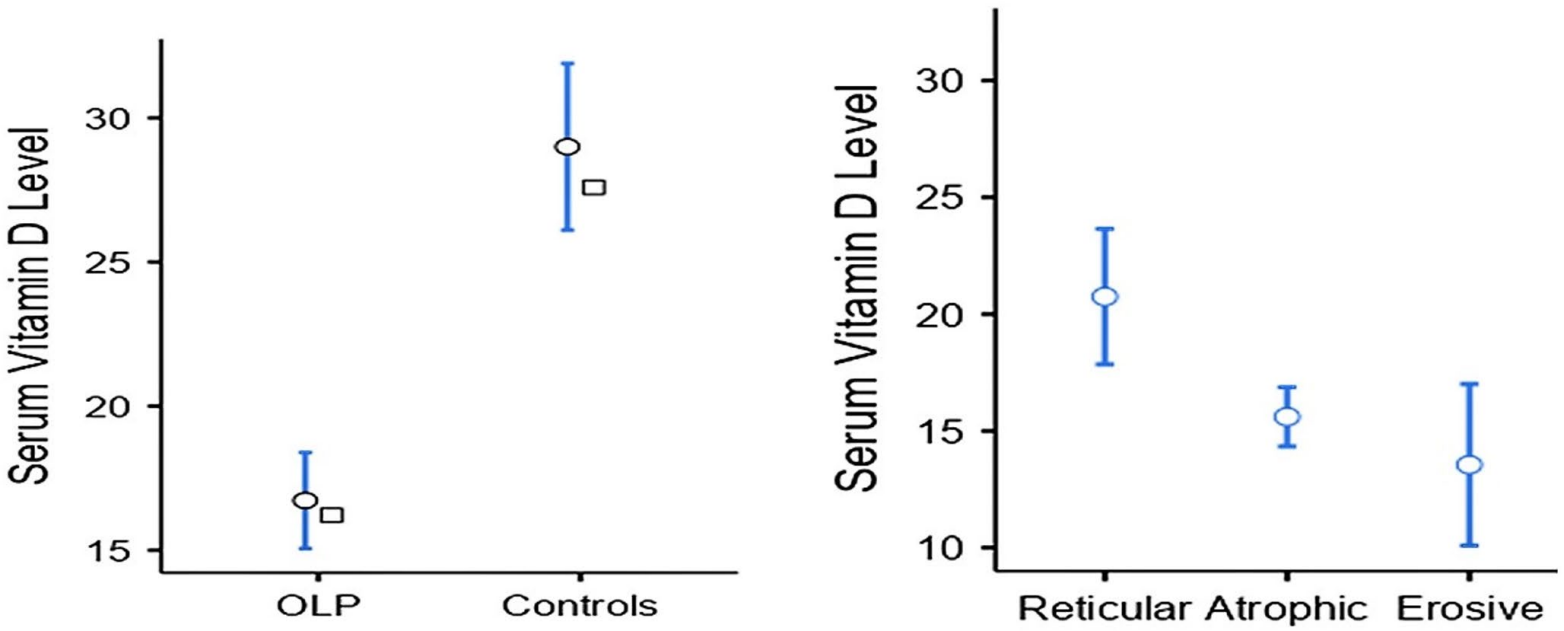
SVDL in OLP patients versus controls and among 3 types of OLP (mean values: OLP = 16.7 ± 5.02, controls = 29 ± 2.16, Reticular OLP = 20.7 ± 4.56, Atrophic OLP = 15.6 ± 1.99 and Erosive OLP = 13.5 ± 5.15)

Regarding SVDL, the mean value ± SD in OLP was 16.7 ± 5.02 ng/ml while in controls was 29 ± 8.73 ng/ml. Mann and Whitney test revealed statistically significant greater values of SVDL in the healthy controls than the OLP group (*P* ≤ 0.001, effect size = 0.818, and CI of -14.8 to -8.00). The Kruskal-Wallis test yielded a statistically significant difference between the 3 forms of OLP (*P* ≤ 0.001 and effect size = 0.616) as demonstrated in Fig. 2. The post hoc analysis revealed that there was a statistically significant difference between reticular and atrophic forms of OLP (*P* = 0.012), as well as reticular and erosive types (*P* = 0.029), while there was no statistically significant difference between atrophic and erosive types of OLP (*P* = 0.344). Similarly, by comparing each type of OLP and controls, there were statistically significant differences between each type and controls (*P* = 0.012, ≤ 0.001, and ≤ 0.001) for reticular, atrophic, and erosive types, respectively. Although lower SVDL was detected in patients with dysplastic lesions (14 ± 4.32) than non-dysplastic lesions (17 ± 5.09), the difference was not statistically significant (*P* = 0.187).

When we compared the two study groups in terms of VD deficiency or sufficiency, there was a statistically significant difference between the two study groups (*P* ≤ 0.001). Furthermore, it was clear that VD deficiency was more pronounced in erosive and atrophic types than reticular types, with a statistically significant difference that reflects the effect of VD deficiency on the severity of the condition (*P* ≤ 0.001) as shown in Table 1.

Statistical analysis was performed between some risk factors for OLP and VD deficiency and it was noticed that only two factors namely, depression and sun exposure significantly affected the number of patients having VD deficiency. As shown in Table 2, both depression and duration of sun exposure significantly affected number of patients having VD deficiency (*P* = 0.001 and 0.027 respectively).

As illustrated in Table 3, binomial logistic regression was performed to test the hypothesized role of SVDL as a risk factor (predictor) for the development of OLP, and the results revealed a statistically significant direct relation (*P* = 0.001) (odds ratio = 1.38; 95%CI = 1.18–1.617). Furthermore, multinomial logistic regression analyzed the correlation between SVDL and different types of OLP, which has also shown a statistically significant direct relation between the progress of the reticular type into more severe forms, namely atrophic and erosive (*P* = 0.022 and 0.004, respectively).

A univariable binary logistic regression analysis was done for determining the risk of OLP from VD deficiency. It revealed that VD deficiency significantly contributed to the development of OLP (*p* < 0.001), with about 26 times increased likelihood of OLP among patients having VD deficiency (OR = 26.156, 95%CI = 7.083–96.593, B coefficient = 3.264, SE = 0.667). This was followed by multivariable analysis to determine the risk of OLP from VD deficiency after controlling for confounding factors which are: age, sex, socioeconomic level, diet habits, duration of sun exposure, and presence of moderate to severe depression, anxiety and stress. The results showed that VD deficiency still significantly contributing to OLP development with an increased risk to about 34 times (AOR = 34.161) after controlling confounders. The multivariable model displayed an accuracy of 85.7% and explained 62.5% of variations in the OLP disease (Table 4). Additionally, Table 5 shows another multivariable logistic regression analysis done to study the effect of SVDL on presence or absence of OLP after adjusting confounders. Similarly, higher SVDL significantly reduce the risk of having OLP (OR = 0.5, 95%CI = 0.33–0.76, *p* = 0.001).

**Table 1:** Participants characteristics

Parameter	Category	OLP *N* = 35 (%)					Control *N* = 35 (%)	*P*-value (OLP vs. Control)
		Reticular (*n* = 12)	Atrophic (*n* = 12)	Erosive (*n* = 11)	*P*-value Between 3 types	Total		
**Age**		53 ± 8.02	52 ± 5.9	51 ± 6.9		52.2 ± 6.8	52.2 ± 6.8	1.000
**Sex**	**Males**	4(16.7)	3(12.5)	5(20.8)		12(34.28)	12(34.28)	1.000
	**Females**	8(17.4)	9(19.6)	6(13)		23(67.72)	23(67.72)	
**Serum Vitamin D**	**VD deficiency**	5(16.1)	12(38.7)	10(32.3)	0.001^a^	27(87.1)	4(12.9)	≤ 0.001^a^
	**VD sufficiency**	7(17.9)	0(0)	1(2.6)		8(20.5)	31(79.5)	
**Residence**	**Urban**	7(21.2)	4(12.1)	4(12.1)		15(42.85)	18(48.6)	0.473^a^
	**Rural**	5(13.5)	8(21.6)	7(18.9)		20(57.15)	17(51.4)	
**Socio-economic status**	**Upper**	0	0	0	0.895	0	0	
	**Upper middle**	3(10.3)	4(13.8)	2(6.9)		9(25.7)	20(57.1)	0.028^b^
	**Middle**	6(23.1)	5(19.2)	5(19.2)		16(45.7)	10(28.6)	
	**lower**	1(14.3)	2(28.6)	3(42.9)		6(17.1)	1(2.9)	
	**Lower middle**	2(25)	1(12.5)	1(12.5)		4(11.4)	4(11.4)	
**DASS-21**								
**Depression**	**Normal**	8(80)	2(20)	0(0)	0.005	10(38.5)	16(61.5)	0.004^b^
	**Mild**	2(66.7)	0(0)	1(33.3)		3(23.1)	10(76.9)	
	**Moderate**	2(16.7)	5(41.7)	5(41.7)		12(60)	8(40)	
	**Severe**	0(0)	5(50)	5(50)		10(90.9)	1(9.1)	
	**Extremely severe**	0	0	0		0	0	
**Anxiety**	**Normal**	4(40)	4(40)	2(20)	0.021	10(34.5)	19(65.5)	0.036^b^
	**Mild**	6(50)	5(41.7)	1(8.3)		12(52.2)	11(47.8)	
	**Moderate**	2(25)	3(37.5)	3(37.5)		8(61.5)	5(38.5)	
	**Severe**	0(0)	0(0)	5(100)		5(100)	0(0)	
	**Extremely severe**	0	0	0		0	0	
**Stress**	**Normal**	4(36.4)	4(36.4)	3(27.3)	0.011	11(36.7)	19(63.3)	0.05^b^
	**Mild**	6(54.5)	5(45.5)	0(0)		11(50)	11(50)	
	**Moderate**	2(25)	3(37.5)	3(37.5)		8(61.5)	5(38.5)	
	**Severe**	0	0	5(100)		5(100)	0(0)	
	**Extremely severe**	0	0	0		0	0	
**Dietary habits**	**Vegetarians**	6(30)	8(40)	6(30)		11(57.1)	11(57.1)	1.000^a^
	**Non-vegetarians**	6(40)	4(26.7)	5(33.3)		24(42.9)	24(42.9)	
**Duration of sun exposure (minutes)**	**≤ 20 m**	8(16)	12(24)	5(10)	0.014	25(71.4)	0(0)	≤ 0.001^a^
	**20–60**	4(20)	0(7)	6(30)		10(28.6)	27(77.1)	
	**≥ 60**	0	0	0		0	8(22.9)	
**Site of the lesion**	**Buccal mucosa**	6(66.7)	2(22.2)	1(11.1)		9 (25.7)		
	**Tongue**	1(14.3)	3(42.9)	3(42.9)		7( 20)		
	**Labial mucosa**	1(20)	2(40)	2(40)		5(14.3)		
	**Hard palate**	1(50)	0(0)	1(50)		2(5.7)		
	**Multisite**	3(25)	5(41.5)	4(33.3)		12 (34.3)		
**Presence of dysplasia**	**Dysplastic OLP**	0	2 (16.7%)	5(45.5%)		7(10%)		
	**Non dysplastic OLP**	12 (100%)	10(83.3%)	6(54.5%)		28(40%)		

a Chi square test

b Fisher exact test

**Table 2:** Serum vitamin D level according characteristics of OLP patients

Parameter	Category	Serum vitamin D level		*P*-value	Control *N* = 35 (%)
		No. of patients (%) with VD sufficiency	No. of patients (%) with VD deficiency		
**Sex**	**Females**	7 (30.4)	16(69.6)	0.216	12(34.28)
	**Males**	1(8.3)	11(91.7)		23(67.72)
**Residence**	**Urban**	4(26.7)	11(73.3)	0.700	18(48.6)
	**Rural**	4(20)	16(80)		17(51.4)
**Socio-economic status**	**Upper**	0	0	0.244	0
	**Upper middle**	2(22.2)	7(77.8)		20(57.1)
	**Middle**	6(37.5)	10(62.5)		10(28.6)
	**lower**	0	6(100)		1(2.9)
	**Lower middle**	0	4(100)		4(11.4)
**DASS-21**				≤ 0.001*	
**Depression**	**Normal**	7(70)	3(30)		16(61.5)
	**Mild**	0	3(100)		10(76.9)
	**Moderate**	1(8.3)	11(91.7)		8(40)
	**Severe**	0	10(100)		1(9.1)
	**Extremely severe**	0	0		0
**Anxiety**	**Normal**	3(30)	7(70)	0.314	19(65.5)
	**Mild**	4(33.3)	8(66.7)		11(47.8)
	**Moderate**	0(0)	8(100)		5(38.5)
	**Severe**	1(20)	4(80)		0(0)
	**Extremely severe**	0	0		0
**Stress**	**Normal**	3(27.3)	8(72.7)	0.312	19(63.3)
	**Mild**	4(36.4)	7(63.6)		11(50)
	**Moderate**	0(0)	8(100)		5(38.5)
	**Severe**	1(20)	4(80)		0(0)
	**Extremely severe**	0	0		
**Dietary habits**	**Vegetarians**	2(18.2 )	9(81.8)	1.000	11(57.1)
	**Non-vegetarians**	6(25)	18(75)		24(42.9)
**Duration of sun exposure (minutes)**	**≤ 20 m**	3(12)	22(88)	0.027*	0(0)
	**20–60**	5(50)	5(50)		27(77.1)
	**≥ 60**	0	0		8(22.9)

*P ≤ 0.05 considered statistically significant

**Table 3:** Regression analysis between serum vitamin D level (Predictor) and occurrence of the disease (outcome)

Binomial logistic regression														
Model Fit Measures							Model Coefficients							
													95% Confidence Intervals	
**Outcome**	**R²**_**cs**_		**Deviance**		**AIC**			**Estimate**	**SE**	**Z**	**p**	**Odds ratio**	**Lower**	**Upper**
**Case** vs. **control**	**0.464**		**53.4**		**57.4**		Intercept	-6.989	1.7	-4.11	0.001	9.22e-4	3.29	0.0258
							**Vitamin D level**	0.324	0.079	4.06	0.001	1.38	1.18	1.617
**Multinomial logistic regression**														
**Model Fit Measures**							**Model Coefficients**							
													**95% Confidence Intervals**	
**Outcome**	**R²**_**cs**_	**Deviance**		**AIC**		**Outcome**		**Estimate**	**SE**	**Z**	**p**	**Odds ratio**	**Lower**	**Upper**
**Atrophic OLP vs. Erosive OLP vs.** **Reticular OLP**	60.9	68.9		0.141		**Atrophic OLP–Reticular OLP**	Intercept	5.66	2.512	2.25	0.024	287.25	0.738	10.58
							**Vitamin D level**	-0.313	0.137	-2.29	0.022	0.731	-0.582	-0.045
						**Erosive OLP–Reticular OLP**	Intercept	7.79	2.76	2.82	0.005	2426	2.379	13.209
							**Vitamin D level**	-0.466	0.161	-2.9	0.004	0.627	-0.782	-0.1506

**Table 4:** Multivariable binary logistic regression analysis for determining the risk of OLP from vitamin D deficiency after controlling for confounding factors

Variable	B coefficient	SE	Adjusted odds ratio (AOR)	95% CI for AOR	*P*-value
Vitamin D deficiency	3.531	0.049	34.161	6.060-192.583	< 0.001*
Age, years	0.038	0.808	1.039	0.944-1.144	0.435
Sex (Female)	0.774	0.789	2.169	0.446-10.563	0.338
Socioeconomic level (Middle)	1.009	0.811	2.742	0.584-12.876	0.201
Diet habits (vegetarian)	0.959	0.711	2.609	0.532-12.784	0.237
Duration of sun exposure (< 20 m)	1.319	1.023	3.741	0.929-15.061	0.063
Moderate-severe depression	0.805	1.161	2.237	0.301-16.608	0.431
Moderate-severe anxiety	0.210	0.049	1.233	0.127-12.005	0.857
Moderate-severe stress	0.212	0.050	1.322	0.129-11.004	0.975

The multivariable model has R^2^ = 62.5%, and an accuracy = 85.7%

*Significant at *p* ≤ 0.05, AOR: adjusted odds ratio, CI: confidence interval, SE: standard error

**Table 5:** Multivariable logistic regression analysis of the effect of SVDL (Predictor) on presence or absence of OLP (outcome) after adjusting confounders

Variable	Odds Ratio (OR)	95% CI	*p*-value
**Vitamin D level**	0.50	0.33–0.76	0.001 *
**Sun exposure (20–60 m)**	3.64	0.50-26.28	0.200
**Higher Socio-economic status**	0.18	0.02–1.31	0.090
**Depression (any)**	0.26	0.03–1.98	0.194
**Age**	1.11	0.97–1.27	0.132
**Male sex**	0.26	0.04–1.71	0.160
**Non-vegetarian diet**	6.29	0.64–61.43	0.114

*Significant at *P* ≤ 0.05

On the other hand, another univariable regression analysis was performed to determine the risk of developing erosive OLP from vitamin D deficiency revealed significant contribution with 4 times increased risk of erosive OLP among patients having vitamin D deficiency (OR = 4.118, 95%CI = 0.440–38.530, B coefficient = 1.415, SE = 1.141). As illustrated in Table 6, another multivariable analysis was performed to study the effect of SVDL on presence or absence of dysplasia in OLP after adjusting confounders. It showed that higher SVDL was significantly associated with lower risk of dysplasia (*p* = 0.041). Likewise, more sun exposure was significantly associated with reduced dysplasia (*p* = 0.045). Socio-economic status, depression, age, sex and diet didn’t show significant associations in this multivariable regression analysis (Table 6).

**Table 6:** Multivariable logistic regression analysis of the effect of SVDL (Predictor) on presence or absence of dysplasia in OLP (outcome) after adjusting confounders

Predictor	Odds Ratio (OR)	95% CI	*p*-value	
**Vitamin D**	1.29	1.01–1.66	0.041*	
**Sun exposure**	14.08	1.07–186.12	0.045*	
**Socio-economic status**	1.46	0.55–3.93	0.45	
**Depression**	1.31	0.51–3.41	0.57	
**Age**	1.11	0.93–1.31	0.25	
**Sex**	3.57	0.33–38.30	0.29	
**Non-vegetarian diet**	6.29	0.64–61.43	0.114	

*Significant at *P* ≤ 0.05

## Discussion

OLP is a chronic inflammatory disease with skin and oral lesions in about 70% of the cases, while 25% of the patients have oral lesions only. It is a highly prevalent disease (0.5–2.2% of whole population) and usually presents with considerable pain and discomfort in atrophic and erosive types [20, 25].

While the papular OLP appears as papules on the oral mucosa, the reticular OLP presents characteristic interlacing white lines, namely Wickham striae. These patterns could change over time. On the other hand, the plaque-like OLP could be clinically indistinguishable from oral leukoplakia. However, oral leukoplakia is usually associated with oral risk factors such as betel quid chewing, cigarette smoking, and alcohol drinking habits which is not the case in the plaque-like OLP. Based on previous literature, being frequently asymptomatic requiring no treatment, the reticular, plaque-like, papular OLP lesions together are referred to as non-erosive OLP (NEOLP) lesions. In addition, both papular and plaque-like OLP usually have concurrent reticular OLP. Accordingly, in this article, these three types of OLP were considered as one group and named reticular OLP.

The atrophic OLP presents as erythematous lesions with fine, peripherally radiating white lines. In more severe cases of OLP, some areas of oral epithelium could separate from the underlying connective tissue resulting in bulla formation which eventually rupture causing erosion and hence, named bullous/erosive OLP. If the erosive OLP lesion has ulcerative areas, it is termed ulcerative OLP. Although sometimes in literature, all these forms including atrophic, erosive, ulcerative, and bullous types are usually grouped as “erosive OLP or EOLP” to be distinguished from its counterpart lesion “NEOLP” as they usually occur with oral symptoms that need treatment. However, in the present study symptomatic EOLP were classified into two types in which lesions showing only erythematous areas were categorized as atrophic OLP, while those presenting with areas of erosion or ulceration or evident bulla were considered as bullous/ erosive. Obviously, the presence of bullae, erosion and ulcerations intensify pain and clinical symptoms and contributes to the debilitation of OLP patients than does the atrophic form. Due to the difference in severity in symptoms and clinical presentation, the atrophic and bullous/ erosive forms were grouped as two distinct types in this study [26].

Additionally, the occurrence of malignant progression of OLP, although debatable, is particularly important [2, 20]. González-Moles et al. reported that the malignant transformation rate was 1.14% for OLP (95% CI = 0.84–1.49) [20]. Although the exact etiopathogenesis of OLP is still ambiguous, its characteristic histopathologic features suggest a crucial role for T-cells in its pathogenesis [3, 25].

VD3 in its active form is a fat-soluble vitamin that is synthesized in the skin with the aid of ultraviolet waves from sunlight [27]. The study of VD3 and its receptors has been trending in recent years due to their involvement in multiple inflammatory, autoimmune, and malignant diseases [28]. Another reason for this trend is the increased prevalence of VD deficiency, which becomes evident due to the increased use of sunscreens and protective clothes in an attempt to protect against skin cancers or due to increased indoor activities with the advancement of technology. Several studies revealed the immune-modulatory, anti-inflammatory, and pro-differentiating properties of VD. In many human malignancies, VD reveals pro-apoptotic properties and prevents angiogenesis and further invasion [12, 27, 29–37].

Evidence from published literature indicates the role of VD in inhibition of pro-inflammatory pathways involving TH-1 and TH-2 cytokines. These anti-inflammatory and immunomodulatory properties suggest a role of VD in OLP pathogenesis [13, 15, 38]. Of interest, VD shows some anti-cancer properties; thus, it is postulated that VD deficiency may be associated with oral cancer or malignant transformation of potentially malignant lesions [13].

Given the current available evidence of the possible association of VD deficiency with the development, progression, and probably malignant transformation of OLP, we aimed to compare SVDL in OLP and healthy controls and to study the relation of SVDL with the severity as well as dysplastic changes of OLP lesions. Regarding SVDL, we found a statistically significant difference between OLP patients and controls (*P* ≤ 0.001). These results are in line with results obtained from 3 studies [4, 11, 25] that reported statistically significant lower SVDL in OLP patients than controls. In accordance with the study done by Seif et al. [39], in which a high percentage of OLP patients showed decreased SVDL, our results revealed that most of the OLP patients (87.1%) had VD deficiency compared to only 12.9% in the control group. Contrarily, Bahramian et al. [14] found no statistically significant difference between OLP and controls (*P* = 0.34).

A recent systematic review [13] pooled results from 4 studies [4, 11, 14, 39] with a total of 206 OLP participants and 170 controls into a meta-analysis. Their results showed that the odds of SVDL in OLP deficiency were higher than those in the control group (odds ratio = 65.95), with a statistically significant difference (*P* = 0.015). In a more recent study by Nosratzehi [40], although he found no statistically significant difference in SVDL between OLP patients and the control group, his results showed that 64% of OLP cases have either VD deficiency or insufficiency. Similarly, Thum-Tyzo et al. [41] reported that 99% of OLP patients have either VD insufficiency or deficiency, which is in line with our results that VD deficiency significantly contributed to the development of OLP (*p* < 0.001), with about 26 times increased likelihood of OLP among patients having VD deficiency (OR = 26.156, 95%CI = 7.083–96.593). Our results, also, showed that VD deficiency still significantly contributing to OLP development with an increased risk to about 34 times (AOR = 34.161) after controlling confounders.

Concerning the relation between SVDL and the severity of OLP, the results revealed statistically significant lower SVDL in each of the erosive and atrophic types of OLP compared to the reticular type and compared to the healthy group. Additionally, the results showed 4 times increased risk of erosive OLP among patients having vitamin D deficiency (OR = 4.118, 95%CI = 0.440–38.530). Similar findings were observed in two studies [25, 42] who found statistically significant lower values for SVDL in symptomatic than asymptomatic OLP. Similarly, Tak et al. [11] showed significantly decreased SVDL in the erosive form of OLP than other types and controls. Another recent study [28] showed the same results with statistical significance (*P* = 0.009). On the contrary, in study by Nosratzehi [40], although SVDL in erosive types was lower than non-erosive types, this difference was not significant.

Additionally, the findings of the present study disclosed non-significant difference in SVDL between dysplastic and non-dysplastic OLP. Yet, upon performing multivariable regression analysis, it revealed that higher SVDL was significantly associated with lower risk of dysplasia (*p* = 0.041). These findings should be deciphered with caution since the number of samples with dysplasia was small (*n* = 7). To our knowledge, no other studies have examined SVDL in dysplastic versus non-dysplastic OLP. Tangarpoor et al. [28] demonstrated a statistically significant lower SVDL in OLP patients than in controls. On the other hand, they found no statistically significant difference between oral squamous cell carcinoma and controls (*P* = 0.96), which could indirectly relates to our results.

To reinforce the results, investigators performed multiple regression analyses to test the hypothesized causal relationship between VD deficiency and the development or progression of OLP, which showed a statistically significant correlation between them even after controlling the confounding factors. Furthermore, other study depicted the improvement in OLP cases after VD administration [15, 16, 43, 44]. This could offer supplementary proof for the role of VD in the pathogenesis and progression of OLP.

In the current investigation, researchers analyzed the correlation between some risk factors that might act as confounders while examining SVDL in OLP patients. Regarding the sex of OLP patients, we found no statistically significant difference between SVDL in male and female OLP patients (*P* = 0.216). These results were in contrast to the study performed on Indian OLP patients, as they found VD deficiency in 77.3% of female patients compared to 58.3% in male patients [4]. However, if we grouped patients with VD deficiency and insufficiency in their study, there would be 87.9% female patients and 86.1% male patients with either VD deficiency or insufficiency, which indicates a non-significant difference as in our study. In another study from Iran, they found that VD deficiency was significantly more pronounced in female OLP patients than females in the control group (*P* = 0.004), while they found a non-significant difference between male OLP patients and males in the control group (*P* = 0.31) [28]. These contradictory outcomes might be explained by the fact that the research took place in different countries with many other contributing factors other than sex.

Evidence from epidemiological studies indicates that exposure to ultraviolet radiation can suppress the initiation and progression of immunological disorders [45]. As VD3 is primarily obtained from exposure to sunlight [27], we compared the interval of sun exposure in OLP and controls as well as its correlation with SVDL. We found a significant correlation between the duration of sun exposure and SVDL in OLP patients (*P* = 0.027). Furthermore, as we compared sun exposure between OLP patients and controls, we found significant difference between them (P 0.001). Likewise, more sun exposure was significantly associated with reduced dysplasia in OLP patients (*p* = 0.045). Our results were consistent with previous study [46], as they found that all (100%) OLP patients with less than 20 min of exposure had either VD deficiency or insufficiency, and the majority of patients with exposure between 20 and 60 had either VD deficiency or insufficiency.

Dietary habit is another factor that may affect SVDL as most of the food rich in VD is from animal sources. However, VD deficiency was found in both vegetarian and non-vegetarian OLP patients with no significant difference (81.8% and 75% respectively). This might be due to the presence of several variables affecting VD absorption and metabolism. These results are consistent with those of Gupta et al. [46] who reported no significant difference between non-vegetarian and vegetarian OLP patients (68.6% and 71.6% respectively).

Concerning socio-economic level, it could affect SVDL in many ways. First, the affordability of particular sorts of sustenance that is rich in VD. Second, the level of education that impacts patient’s awareness about their physical and oral health; and third, the affordability of regular visits to a dentist or physician. Additionally, some people live in crowded places with reduced sun penetration [47]. Despite the aforementioned reasons, we found no significant difference in SVDL among different socio-economic classes of OLP patients (*P* = 0.244). A possible explanation is the excessive use of sunscreens aimed at protecting against skin cancer in higher socioeconomic classes. In addition, the variability of technological devices and a more luxurious lifestyle may limit outdoor activities. Even some professions were directed towards working from home during the COVID-19 pandemic.

Psychological factors, including depression, anxiety and stress, have been linked to OLP as it may alter both the endocrine and immunologic responses in OLP patients [17, 48]. Accordingly, these parameters were examined using DASS-21 scores [18]. We found that there was a significant elevation in stress, anxiety, and depression scores in OLP patients compared to controls (*P* = 0.004, 0.036, and 0.05, respectively). Although VD deficiency was more pronounced in OLP patients presenting with depression, anxiety, and stress than VD sufficiency, there was no statistically significant correlation between these factors and SVDL among different types of OLP. These results were in agreement with Shaw et al. who detected significantly higher depression, anxiety and stress scores in OLP [49].

As for the place of residence, urbanization has led to an increased amount of stressors, such as environmental pollution, overcrowding, and increased life challenges compared to rural areas. Additionally, atmospheric pollution could reduce VD synthesis as it results in decreased penetration of solar radiation. Nevertheless, people in rural areas are also prone to VD deficiency, as villagers usually depend on Phytates in their diet, which are easily available and affordable. Phytates cause increased catabolism of VD with a resultant low SVDL [50, 51]. So, it was concluded that people living in either rural or urban areas can be susceptible to VD deficiency. In that sense, when we analyzed SVDL in OLP patients living in either rural or urban areas, results revealed no significant difference in SVDL between them (*P* = 0.7),

The available evidence suggests a notable association between vitamin D deficiency and various immunological and inflammatory disorders, including Oral Lichen Planus (OLP) [21–24]. On the other hand, research has demonstrated the anti-inflammatory properties of vitamin D, attributed to its capacity to modulate the immune response through several mechanisms. These include the modulation of macrophage activity, induction of dendritic cell maturation, and suppression of pro-inflammatory TH-1 and TH-2 pathways. Additionally, previous studies have indicated that increased dietary intake of vitamin D is associated with a reduced incidence of inflammatory diseases [25, 26]. Furthermore, in several malignancies, vitamin D exhibits pro-apoptotic properties, suppresses invasion, and inhibits angiogenesis [17, 25–28].

In light of these findings, our results reinforce the significant association between vitamin D deficiency and the presence and severity of OLP, particularly in atrophic and erosive forms, as well as in cases exhibiting dysplasia. These observations underscore the potential clinical relevance of monitoring and managing serum vitamin D levels in OLP patients to prevent disease progression. Given the chronic nature of OLP, particularly in its more painful and debilitating forms, reduced engagement in outdoor activities and diminished sun exposure may contribute to further exacerbation of the condition. Thus, addressing vitamin D deficiency in OLP patients could be crucial for mitigating disease progression and improving patient outcomes.

### Strength, limitations and future recommendations

The current study has addressed an important topic as it investigated two prevalent conditions, OLP and VD deficiency, both of which are common in many populations, especially in developing countries, including Egypt. Moreover, it considered many of the possible confounders, such as age, sex, childhood living condition, duration of sun exposure, socioeconomic level, depression, anxiety, stress and dietary habits. Furthermore, there is rarity in studies that evaluate SVDL in dysplastic versus non-dysplastic cases. Nevertheless, some limitations do exist such as the small sample size, which is particularly true for the number of dysplastic cases and could affect the reliability of results. Consequently, there is not enough evidence to suggest that VD deficiency contributes to the malignant transformation of OLP. However, if a larger sample size was examined, there may be different results. There is, also, lack of data about basal metabolic rate, serum parathyroid, and calcium levels. Therefore, we recommend that further experiments should have a greater sample size to confirm our results. Extra research is required to evaluate VDRs in OLP patients using molecular and histochemical studies. Additionally, future studies should investigate the influence of VD supplementation on the initiation and progression and probably the management of OLP in different seasons. Finally, we hope that this study can raise awareness about the importance of VD as a contributing factor in some immunologic diseases, including OLP.

## Conclusion

The present study could corroborate the evidence from previous reports that VD deficiency is a potential risk factor affecting the initiation of OLP and validate its association with more severe forms of the disease, including the incidence of epithelial dysplasia. However, further well-designed studies with larger sample sizes are still required to substantiate the current study’s findings.

## Electronic supplementary material

Below is the link to the electronic supplementary material.

Supplementary Material 1

## Data Availability

The data that support the findings of this study are not openly available due to reasons of sensitivity and are available from the corresponding author upon reasonable request.
