# Supplementary material for: The role of vitamin D deficiency in the development and severity of oral lichen planus: a case-control study
Source: Clin Oral Investig. 2025 May 30;29(6):320. doi: 10.1007/s00784-025-06398-y (PMC12125093; doi:10.1007/s00784-025-06398-y)
Supplement: Supplementary file 1 — Supplementary Material 1 [file 784_2025_6398_MOESM1_ESM.docx]

**Table (suppl.1): Comparison of serum vitamin D level of OLP patients versus controls**

| **Variables** | **Participants** | **N** | **Mean± SD** | **SE** | **Mann and Whitney test (non-parametric)** | | | | | |
| --- | --- | --- | --- | --- | --- | --- | --- | --- | --- | --- |
|  |  |  |  |  | **Statistics** | **Mean difference** | **P-value** | **95% CI** | | **Effect size** |
|  |  |  |  |  | 112 | **-11.00** | ≤0.001 | **Lower** | **Upper** |  |
| **Serum Vitamin D level** | OLP case | 35 | 16.7±5.02 | 0.849 |  |  |  | -14.8 | -8.00 | 0.818 |
|  | Controls | 35 | 29±2.16 | 1.48 |  |  |  |  |  |  |
|  |  |  |  |  | **Student’s t test (parametric)** | | | | | |
|  | **Participants** | **N** | **Mean± SD** | **SE** | **Statistics** | **Mean difference** | **P-value** | **95% CI** | | **Effect size** |
|  |  |  |  |  | -1.35 | -2.83 | 0.187 | Lower | Upper | -0.570 |
|  | Dysplastic OLP | 7 | 14.5…4.32 | 1.63 |  |  |  | -7.10 | 1.44 |  |
|  | Non-dysplastic OLP | 28 | 17.3…5.09 | 0.963 |  |  |  |  |  |  |
|  | **Participants** | **N** | **Mean± SD** | **SE** | **Kruskal-Wallis (non-parametric)** | | | | | |
|  |  |  |  |  | **χ²** | **P-value** | | **Effect size (ε²)** | | |
|  | Reticular OLP | 12 | 20.7±4.56 | 1.316 | 42.5 | ≤0.001 | | 0.616 | | |
|  | Atrophic OLP | 12 | 15.6±1.99 | 0.576 |  |  |  |  |  |  |
|  | Erosive OLP | 11 | 13.5±5.15 | 1.551 |  |  |  |  |  |  |
|  | Controls | 35 | 29±8.73 | 1.476 |  |  |  |  |  |  |

**Table (suppl.2): Dwass-Steel-Critchlow-Fligner pairwise comparisons- Serum Vitamin D level**

|  |  | **W** | **P-value** |
| --- | --- | --- | --- |
| **Reticular OLP** | **Atrophic OLP** | -4.33 | 0.012 |
| **Reticular OLP** | **Erosive OLP** | -3.92 | 0.029 |
| **Atrophic OLP** | **Erosive OLP** | -2.35 | 0.344 |
| **Reticular OLP** | **Controls** | 4.31 | 0.012 |
| **Atrophic OLP** | **Controls** | 6.99 | 0.001 |
| **Erosive OLP** | **Controls** | 6.32 | 0.001 |

**Table (suppl.3): Average serum vitamin D level in each group**

| **Group Descriptives** | | | | | | | | | | | | | | | | | | | | | | | |
| --- | --- | --- | --- | --- | --- | --- | --- | --- | --- | --- | --- | --- | --- | --- | --- | --- | --- | --- | --- | --- | --- | --- | --- |
|  | | **Group** | | **N** | | | **Mean** | | | | | **Median** | | | | **SD** | | | | **SE** | | | |
| **Vitamin D level** |  | **OLP** |  | **35** |  | | **16.7** | | | |  | **16.2** | | |  | **5.02** | | |  | **0.849** | | |  |
|  | | **Controls** |  | **35** |  | | **29.0** | | | |  | **27.6** | | |  | **8.73** | | |  | **1.48** | | |  |
|  | | | | | | | | | | | | | | | | | | | | | | | |
|  | | **participants** | | | | | | **N** | | **Mean** | | | | **SD** | | | | **SE** | | | |  |  |
| **Vitamin D level** |  | **Reticular OLP** | | | |  | | **12** |  | **20.7** | | |  | **4.56** | | |  | **1.316** | | |  |  |  |
|  |  | **Atrophic OLP** | | | |  | | **12** |  | **15.6** | | |  | **1.99** | | |  | **0.576** | | |  |  |  |
|  |  | **Erosive OLP** | | | |  | | **11** |  | **13.5** | | |  | **5.15** | | |  | **1.551** | | |  |  |  |
|  |  | **Controls** | | | |  | | **35** |  | **29.0** | | |  | **8.73** | | |  | **1.476** | | |  |  |  |
|  | | | | | | | | | | | | | | | | | | | | | |  |  |


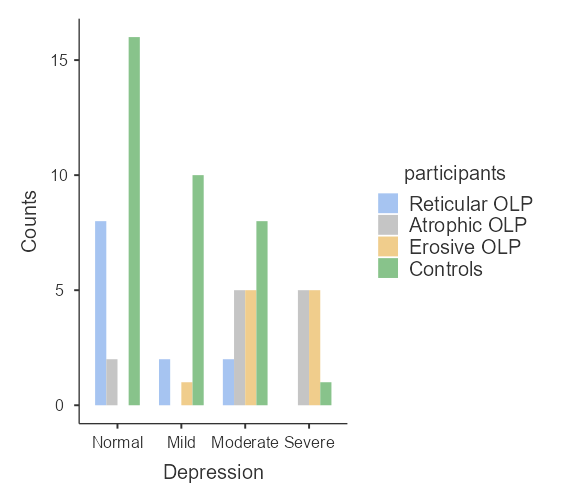


**Figure (suppl.1): Depression scores in 3 types of OLP and Controls**


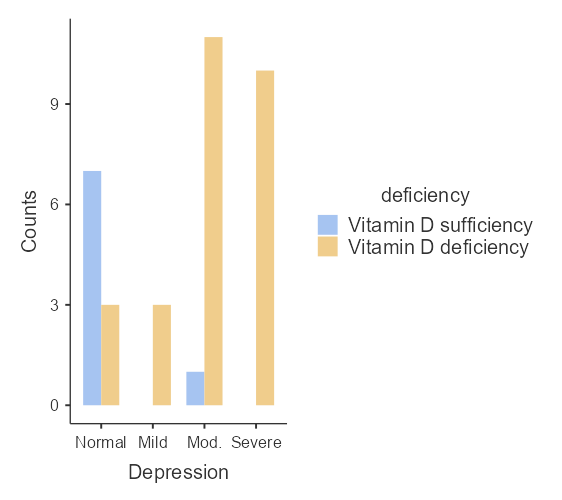


**Figure (suppl.2): Correlation between depression and vitamin D deficiency**


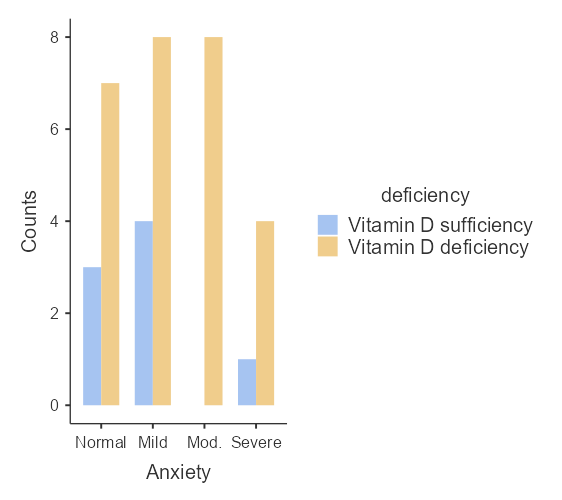


**Figure (suppl.3): Correlation between anxiety and vitamin D deficiency**
